# Supplementary material for: High-Throughput Gel Microbeads as Incubators for Bacterial Competition Study
Source: Micromachines (Basel). 2023 Mar 12;14(3):645. doi: 10.3390/mi14030645 (PMC10058504; doi:10.3390/mi14030645)
Supplement: Supplementary file 1 [file micromachines-14-00645-s001.zip › micromachines-2259178-supplementary.pdf]

Supplementary Materials

High-Throughput Gel Microbeads as Incubators for Bacterial Competition Study

Trang Anh Nguyen-Le, Xinne Zhao, Michael Bachmann, Philip Ruelens, J. Arjan G. M. de Visser and Larysa Baraban *

Supplementary note 1: Bacteria preparation

Bacteria stock is pre-made by transferring a single colony of desired strain on agar petri dish into a sterilized flask containing fresh LB broth and incubating until mid-exponential phase. The stock is mixed with 70% glycerol and kept in a freezer at -80°C for long-term storage. Once a month, a small amount of frozen stock is scraped off with a sterile pipette tip, transferred to a sterile flask containing 50 mL of culture medium, and incubated overnight at 37°C for recovery. For each experiment, we transfer 100 µL of this activated culture to a sterile flask containing both culture medium and nutrients and incubate the solution in a thermostat shaker at 170 rpm until reaching the mid-exponential phase. The liquid batch is stored in the fridge at 4°C for temporary usage.

Supplementary Tables and Figures:

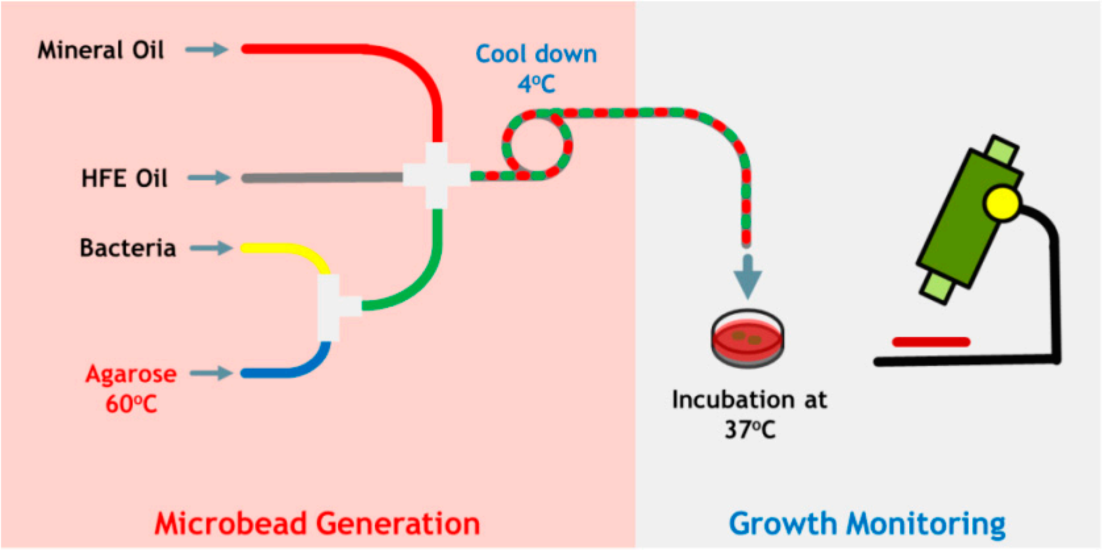

Figure S1: Experimental setup for studying colonial growth of E. coli inside agarose microbeads. Bacterial solution was quickly mixed with hot agarose solution (60 °C) at T-junction. E. coli populations were then encapsulated in aqueous droplets at the cross-junction and entered the reserved coil. The coil was later cooled down to 4°C to induce gelation. A sterilized 3.5-cm petri dish was prepared for collection of microbeads. The microbeads were covered with bio-graded mineral oil and examined by conventional fluorescence microscope.

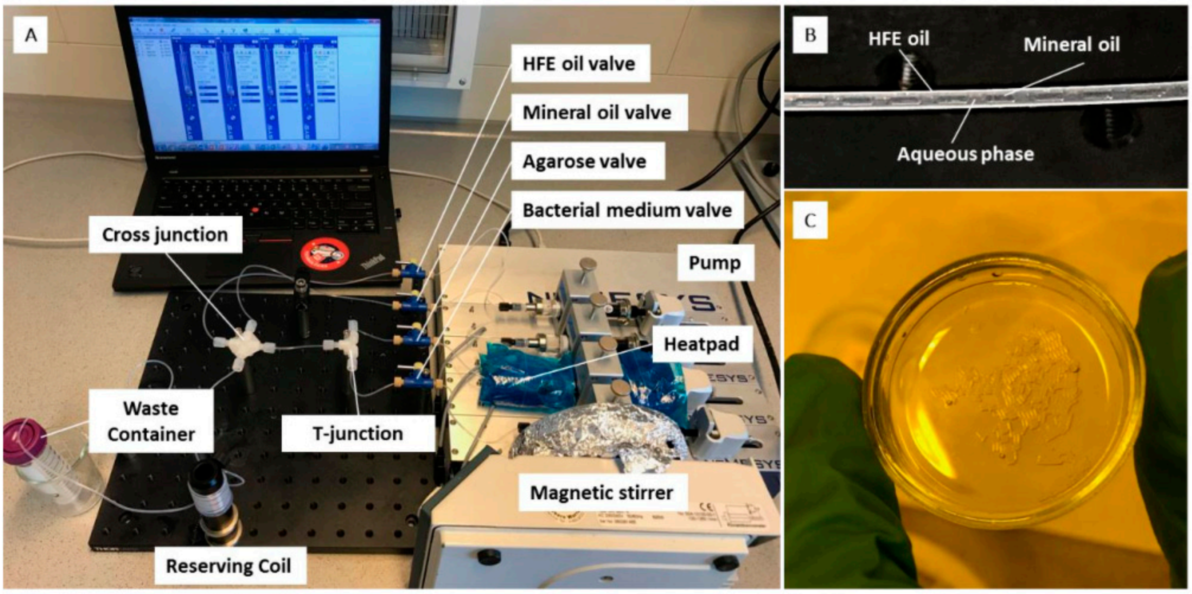

Figure S2: Experimental setup for encapsulation of bacteria in agarose microbeads (A) a fluidic network with one T-junction for mixing of bacterial medium and agarose solution and one cross-junction for droplet generation (B) droplet train with flow rate of 1/7/3 mL/h for HFE/mineral oil/aqueous phase (C) after gelation microbeads were collected in a petri dish for examination

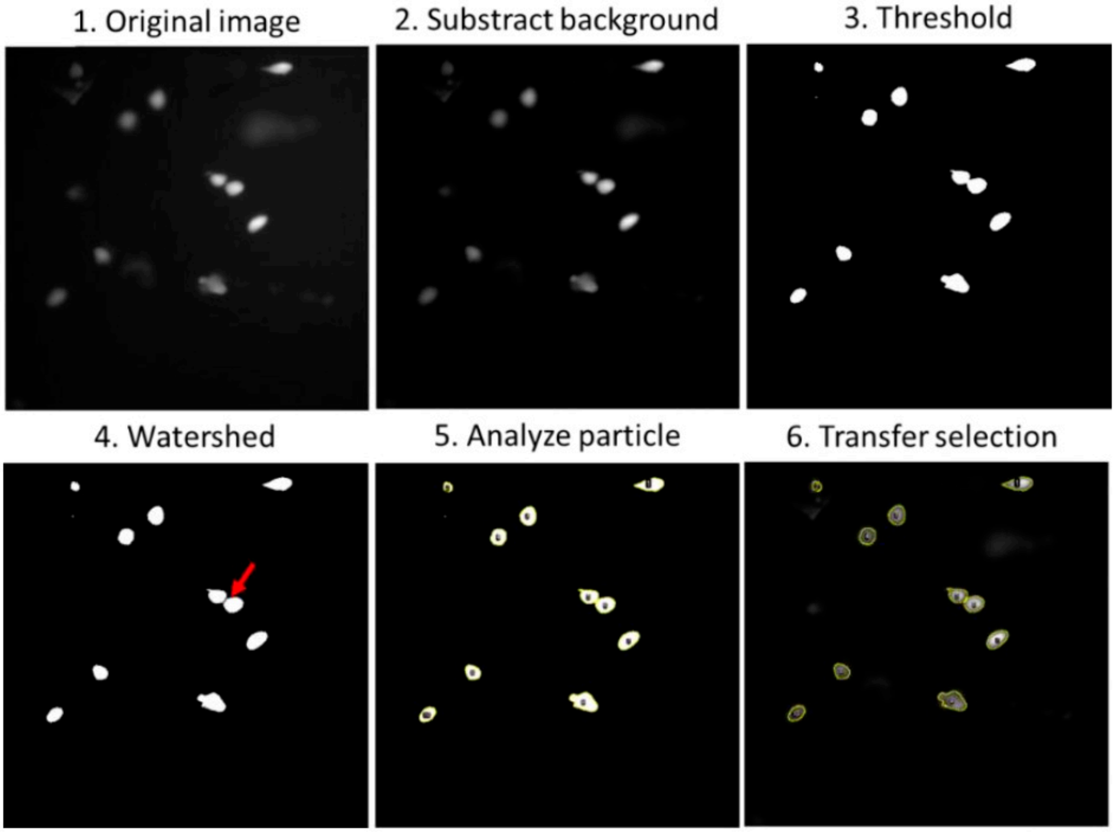

Figure S3: Image processing for analysis of microcolonies in gel beads. The red arrow shows the difference between the results in step 3 and 4. Firstly, the background noise was subtracted from the original fluorescence picture (2). The picture was then converted to a binary image (3) and treated with the watershed algorithm to eliminate potential overlapping (4). Using "Analyze particle" package from ImageJ, the boundary of each colony was drawn (5). The boundaries were transferred to the subtracted picture (2) for analysis of fluorescent intensity (6).

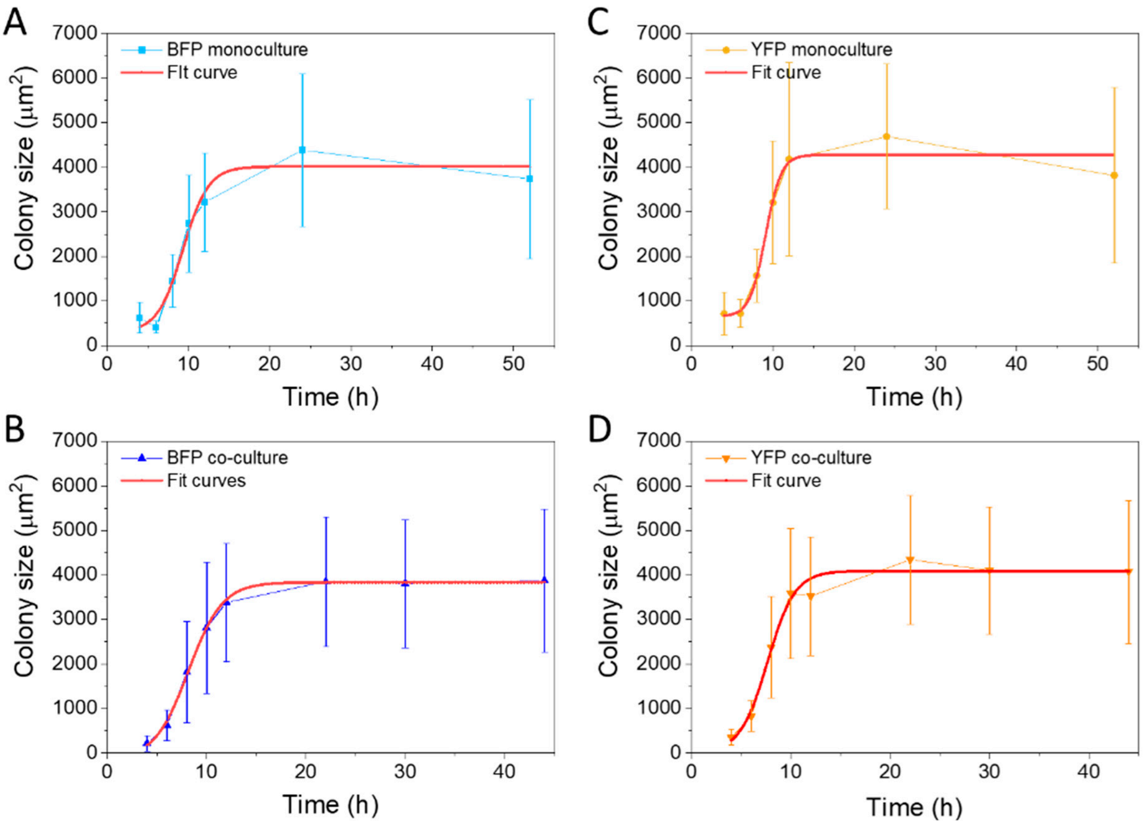

Figure S4: Fit curves of colony size: (A) E. coli BFP monoculture, (B) E. coli BFP co-culture, (C) E. coli YFP monoculture and (D) E. coli YFP co-culture. The fitting is based on the experimental results (monoculture from 4 to 52 hours and co-culture from 4 to 44 hours) with sigmoidal growth curve function.

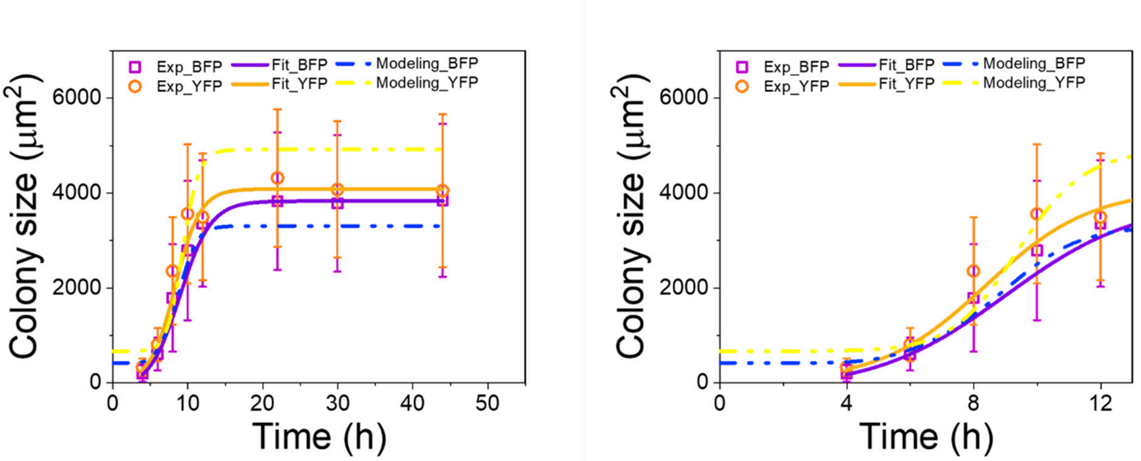

Figure S5: Comparison of experimental co-culture data points with co-culture fit curves and modeling growth curves of co-culture E. coli BFP and E. coli YFP. (Left) 0-52 hours (Right) 0-12 hours.

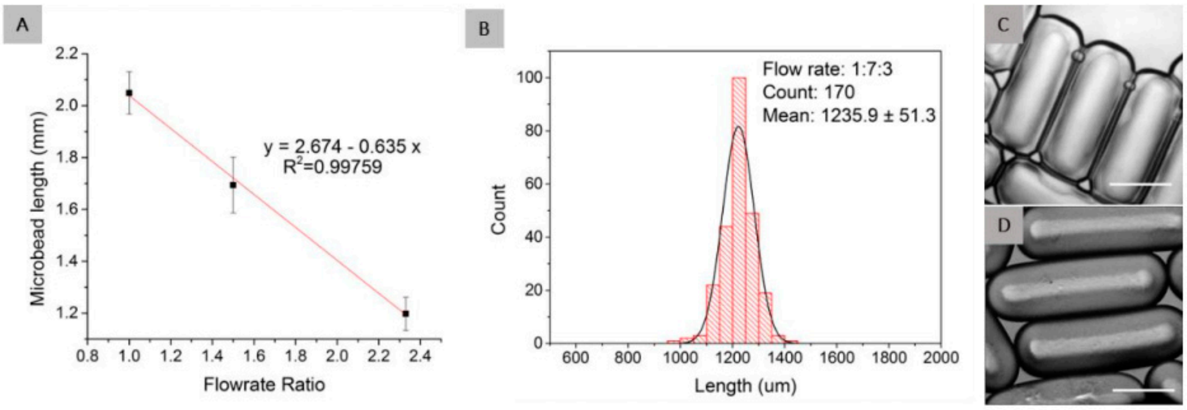

Figure S6: Microbeads generated in the final setup (A) effect of flow rate ratio on microbead length (the flow rate ratio was for mineral oil/aqueous phase) (B) microbead size at the chosen flow rates 1:7:3 (C) microbeads generated by the cross junction in the final setup (D) by T-junction in the prototype (Scale bar: 500μm)

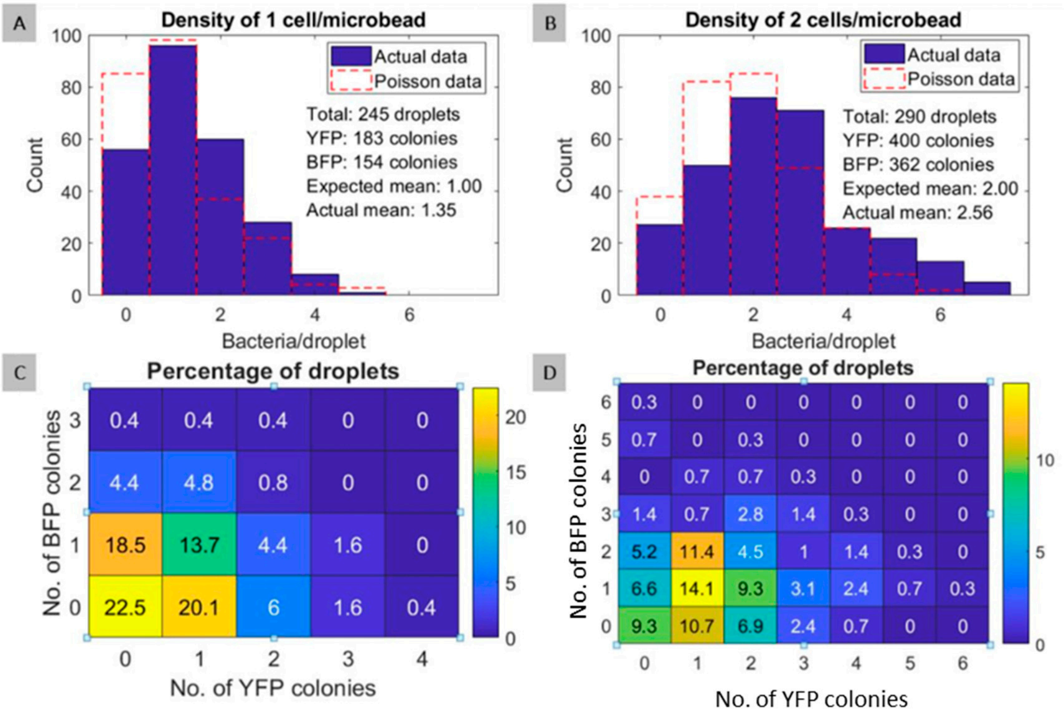

Figure S7: Distribution of bacteria colonies in microbeads at low inoculation density. (A,B) Histograms show the total number of colonies counted for each microbead when inoculated with (A) 1 cell/droplet and (B) 2 cell/droplet. The dashed bars represent the expected Poisson distribution for each situation. (C,D) Heatmaps show the percentage of microbeads that contained a specific number of E. coli YFP and E. coli BFP colonies when inoculated with (C) 1 cell/droplet and (D) 2 cell/droplet. Color map shows the filling rate.

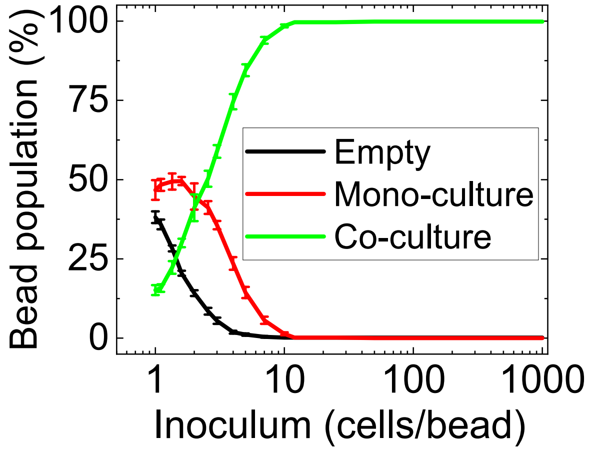

Figure S8: Monte-Carlo simulation for bacteria distribution in gel beads. All simulations were performed on MATLAB R2020b. 500 empty sets of data were generated equivalent to the initial 500 gel beads. The average percentage of each type of bead from ten simulations is reported for each inoculum size as follows. The error bars present the standard deviation of ten simulations.

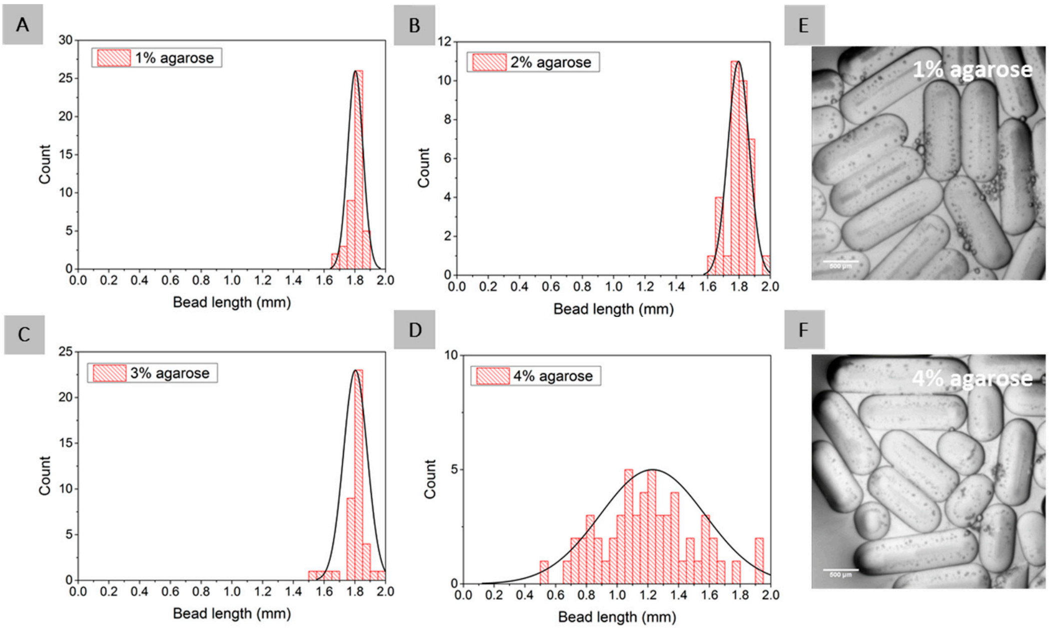

Figure S9: Effect of agarose concentration on microbead size (A-D) Histogram of microbead length with agarose concentration ranging from 1% to 4%, (E) microscope images of microbead generated with 1% agarose, (F) with 4% agarose.

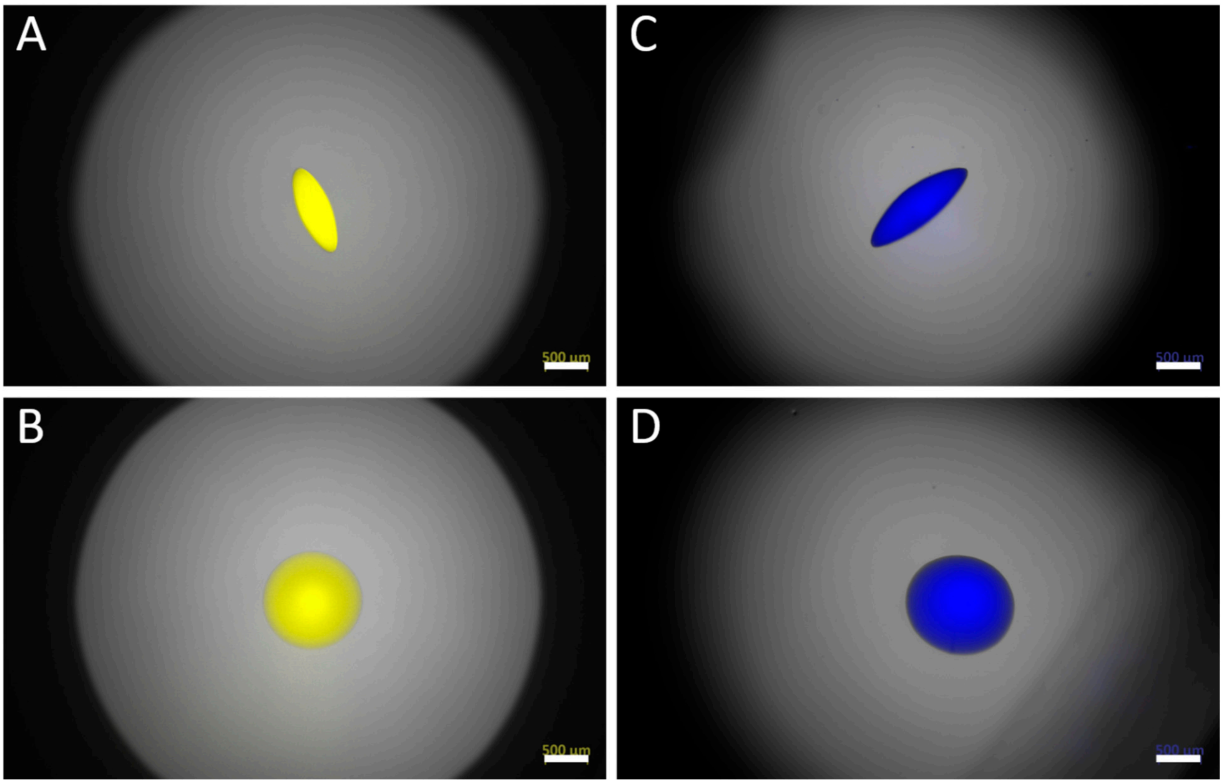

Figure S10: Colony shape of monoculture E. coli BFP and E. coli YFP formed in and on the surface of Agar plate. (A) monoculture E. coli YFP formed in Agar plate; (B) monoculture E. coli YFP

formed on Agar plate surface; (C) monoculture E. coli BFP formed in Agar plate; (D) monoculture E. coli YFP formed on Agar plate surface. Error bar is 500 µm.

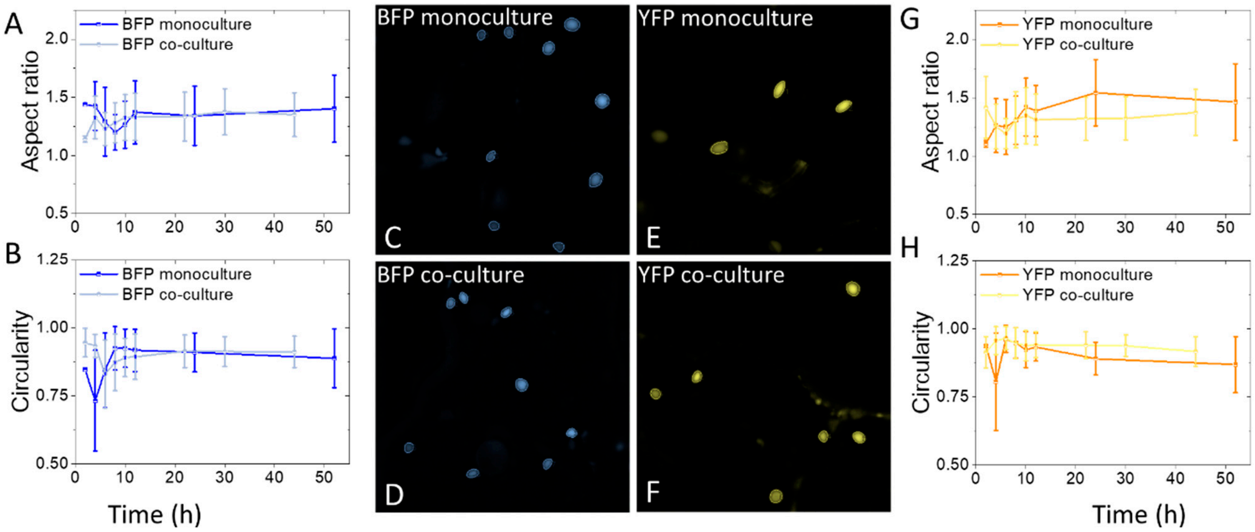

Figure S11: Comparison colony shape between two strains of E. coli in monoculture and co-culture: **(A)** aspect ratio and **(B)** circularity of E. coli BFP monoculture and E. coli BFP co-culture and microscopic images of **(C)** E. coli BFP monoculture and **(D)** E. coli BFP co-culture (with E. coli YFP) in agarose microbeads after 20 hours; microscopic images of **(E)** E. coli YFP monoculture and **(F)** E. coli YFP co-culture (with E. coli BFP) in agarose microbeads after 20 hours and **(G)** aspect ratio and **(H)** circularity of E. coli YFP monoculture and E. coli YFP co-culture.

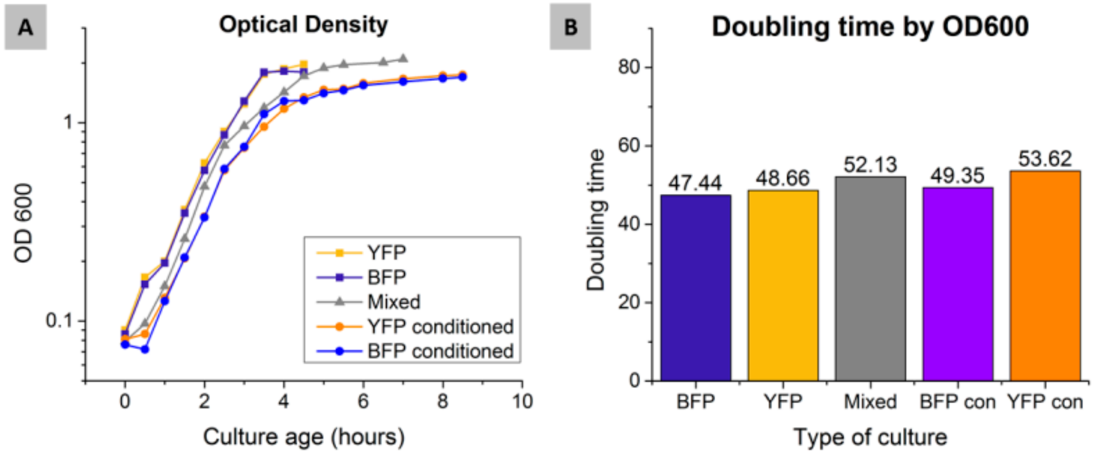

Figure S12: Growth behavior of E. coli YFP and E. coli BFP in liquid medium measured by optical density. (A) Growth curve constructed by OD 600 measurements, note that the y-axis is in log scale. Since OD 600 and cell density have a linear relationship, the y-axis in the original value will not affect the analysis. (B) Doubling time of bacteria in each condition calculated by linear fitting 8 data points of each curve in the exponential phase.

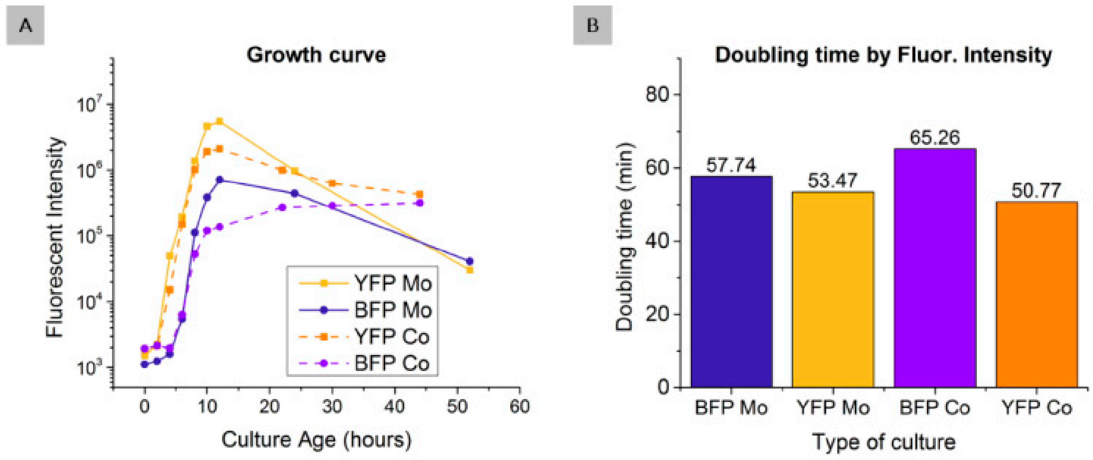

Figure S13: Growth behavior of E. coli YFP and E. coli BFP in agarose microbead measured by fluorescence microscope. (A) Growth curves collected from Figure 37C-D. (B) Doubling time of bacteria in each condition was calculated by linear fitting 4 data points from 4 to 10 hours of each curve.

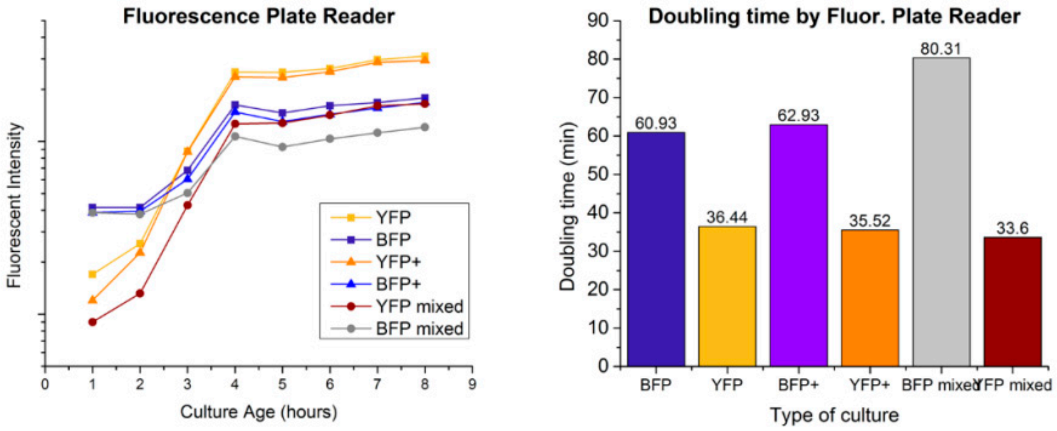

Figure S14: Growth dynamic and doubling time obtained by Tecan Infinite 200 microwell plate reader The bacteria were cultivated in 3 conditions: individually (YFP and BFP), with ratio 9:1 (YFP+ and BFP+) and ratio 1:1 (YFP and BFP mixed). In all situations, E. coli BFP grew much slower than E. coli YFP. The doubling time increased with the number of E. coli YFP inoculated.

Table S1: Effect of initial density on final colony area

	E. coli BFP measured colony area (μm²)				E. coli YFP measured colony area (μm²)			
**Inoculum**	**mean**	**median**	**std**	**count**	**mean**	**median**	**std**	**count**
**1**	9553.4	9543.7	3689.0	59	10338.6	9543.7	4102.0	71
**2**	3837.5	3952.6	1628.7	119	4155.2	4075.5	1547.8	131
**10**	1552.3	1321.0	950.0	107	1919.6	1868.8	933.3	136
**100**	365.9	297.2	232.4	98	150.8	304.6	201.6	115
**500**	143.3	122.9	92.4	137	147.2	125.4	91.3	134
